# MicroRNA-21 plays an oncogenic role by targeting FOXO1 and activating the PI3K/AKT pathway in diffuse large B-cell lymphoma

**DOI:** 10.18632/oncotarget.3729

**Published:** 2015-03-30

**Authors:** Heounjeong Go, Ji-Young Jang, Pil-Jong Kim, Young-Goo Kim, Soo Jeong Nam, Jin Ho Paik, Tae Min Kim, Dae Seog Heo, Chul-Woo Kim, Yoon Kyung Jeon

**Affiliations:** ^1^ Department of Pathology, Seoul National University Hospital, Seoul National University College of Medicine, Seoul, South Korea; ^2^ The Tumor Immunity Medical Research Center, Cancer Research Institute, Seoul National University College of Medicine, Seoul, South Korea; ^3^ Department of Pathology, Asan Medical Center, University of Ulsan College of Medicine, Seoul, South Korea; ^4^ The Tumor Microenvironment Global Core Research Center, Seoul National University, Seoul, South Korea; ^5^ Biomedical Knowledge Engineering Laboratory, Seoul National University School of Denistry, Seoul, South Korea; ^6^ Department of Pathology, Seoul National University Bundang Hospital, Seoul National University College of Medicine, Seongnam, Gyeonggi, South Korea; ^7^ Department of Internal Medicine, Seoul National University Hospital, Seoul National University College of Medicine, Seoul, South Korea

**Keywords:** diffuse large B-cell lymphoma, miR-21, miR-17-92 cluster, miR-155, FOXO1

## Abstract

The prognostic implications of miR-21, miR-17-92 and miR-155 were evaluated in diffuse large B-cell lymphoma (DLBCL) patients, and novel mechanism by which miR-21 contributes to the oncogenesis of DLBCL by regulating FOXO1 and PI3K/AKT/mTOR pathway was investigated. The expressions of miR-21, miR-17-92 and miR-155 measured by quantitative reverse-transcription-PCR were significantly up-regulated in DLBCL tissues (n=200) compared to control tonsils (*P=*0.012, *P=*0.001 and *P*<0.0001). Overexpression of miR-21 and miR-17-92 was significantly associated with shorter progression-free survival (*P=*0.003 and *P=*0.014) and overall survival (*P=*0.004 and *P=*0.012). High miR-21 was an independent prognostic factor in DLBCL patients treated with rituximab-combined chemotherapy. MiR-21 level was inversely correlated with the levels of FOXO1 and PTEN in DLBCL cell lines. Reporter-gene assay showed that miR-21 directly targeted and suppressed the FOXO1 expression, and subsequently inhibited Bim transcription in DLBCL cells. MiR-21 also down-regulated PTEN expression and consequently activated the PI3K/AKT/mTOR pathway, which further decreased FOXO1 expression. Moreover, miR-21 inhibitor suppressed the expression and activity of MDR1, thereby sensitizing DLBCL cells to doxorubicin. These data demonstrated that miR-21 plays an important oncogenic role in DLBCL by modulating the PI3K/AKT/mTOR/FOXO1 pathway at multiple levels resulting in strong prognostic implication. Therefore, targeting miR-21 may have therapeutic relevance in DLBCL.

## INTRODUCTION

Diffuse large B-cell lymphoma (DLBCL) is the most common type of non-Hodgkin lymphoma, and it accounts for 30-40% of the newly diagnosed cases of non-Hodgkin lymphoma [1]. DLBCL is a highly heterogeneous group with variable clinical features, molecular genetic alterations, therapeutic responsiveness levels, and prognoses [2]. DLBCL can be classified into subgroups according to the cell of origin, genetic aberrations, microenvironmental signature, and dysregulated signaling pathways [1, 3, 4]. Typically, activated B-cell-like (ABC) DLBCL shows chronic active B-cell receptor (BCR) signaling and MYD88 signaling due to recurrent genetic mutations involving *CD79A/B* and *MYD88*, which eventually leads to constitutive activation of NF-κB [5]. BCR and MYD88 signaling have also been shown to activate the PI3K/AKT and JAK/STAT pathways to promote cell survival in cooperation with the NF-κB pathway [5, 6]. In contrast, germinal center B-cell-like (GCB) DLBCLs have been shown to be addicted to the oncogenic activation of the PI3K/AKT pathway [7]. Moreover, AKT activation is associated with poor prognosis of DLBCL patients [8]. However, the mechanism underlying the activation of PI3K/AKT pathway and its oncogenic role in DLBCL remain unclear.

MiRNAs are small non-coding RNAs of 20-22 nucleotides implicated in a variety of physiological and pathological processes [9]. In the hematolymphoid system, miRNAs play a pleiotropic role and are involved in B-cell differentiation and malignant transformation. Several miRNAs also regulate oncogenic or tumor-suppressive pathways, such as the NF-κB or BCR signaling, in lymphoma [10, 11]. MiR-21, the miR-17-92 cluster (miR-17-92 hereafter) and miR-155 are well-known oncogenic miRNAs, which mostly target tumor-suppressive molecules in many cancers [12]. Overexpression of miR-21, miR-17-92 and miR-155 were observed in several lymphomas derived from B-cells, T-cells or NK-cells, showing diagnostic, prognostic, and therapeutic potentials [10, 13-15].

Specifically, miR-21 played an important role in pre-B-cell lymphomagenesis, and inactivation of miR-21 caused the regression of tumors via apoptosis and cell-cycle arrest in a mouse model [16]. MiR-21 was also reported to regulate the chemosensitivity of DLBCL cells [17]. MiR-17-92 was the first miRNA identified as dysregulated in DLBCL [18], and was demonstrated to induce B-cell leukemia in concert with MYC [19, 20]. MiR-155 directly targets SMAD5 and to help lymphoma cells escape from TGF-β-mediated growth-inhibition [21].

However, the status of miR-21, miR-17-92 and miR-155 and their clinicopathological implications are not fully elucidated in patients with DLBCL. Moreover, the mechanisms by which they contribute to the pathogenesis of human DLBCLs are not completely understood. Thus, in this study, we analyzed the association of the miR-21, miR-17-92 and miR-155 expression with the clinicopathological features and prognosis of patients with DLBCL. Furthermore, we investigated the role of miR-21 in the modulation of the PI3K/AKT pathway in DLBCL cells, and we discovered that FOXO1 is a novel direct target of miR-21.

## RESULTS

### MiR-21, miR-17-92 and miR-155 expression levels in DLBCL patients and their associations with the clinicopathological features

**Figure 1:**
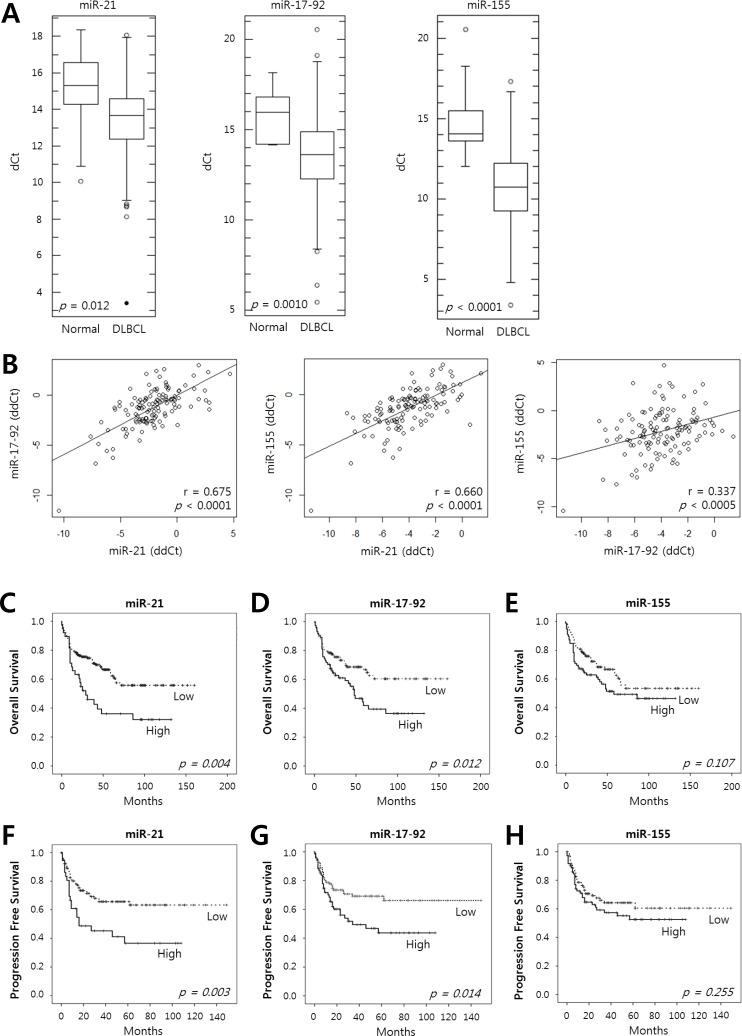
Expression of miR-21, the miR-17-92 cluster and miR-155 in tumor tissues of DLBCL patients and their prognostic implications **A**, The levels of expression of miR-21, the miR-17-92 cluster and miR-155 were evaluated in the tumor tissues of DLBCL patients (n = 200) and in normal tonsils as a control (n = 11) using qRT-PCR. Box-and-whisker plots demonstrate the levels of expression of miR-21, the miR-17-92 cluster, and miR-155 as dCt (= Ct_(miRNA)_ - Ct_(U6)_) values in the DLBCL tissues compared with those in normal tonsils. A lower dCt implies a higher relative level of miRNA. The differences in the miRNA levels of the DLBCL and normal group were evaluated using Student's t-test. **B**, The relative expression levels of the miRNAs were calculated as ddCt (= dCt_(case)_ - mean dCt_(control)_). The diagrams show the correlation of the relative expression levels of miR-21, the miR-17-92 cluster and miR-155 determined using Pearson's correlation analysis (r, correlation coefficient). Kaplan-Meier curves obtained using the log-rank test show **C**-**E**, the overall survival and **F**-**H**, the progression-free survival of patients with DLBCLs with high *vs.* low relative expression levels of miR-21, the miR-17-92 cluster, and miR-155.

The expression levels of miR-21, miR-17-92 and miR-155 in the DLBCL tissues determined using quantitative reverse-transcription polymerase chain reaction (qRT-PCR) were significantly up-regulated and showed lower dCt values compared to those of controls (*P* = 0.012, *P* = 0.001, *P* <0.0001, respectively) (Fig. 1A). The expression levels of these miRNAs relative to those of normal tonsils, as represented by ddCt values, showed significant positive correlations with each other (miR-21 *vs*. miR-17-92, *P* <0.0001; miR-21 *vs*. miR-155, *P* < 0.0001; miR-17-92 *vs.* miR-155, *P* <0.0005) (Fig. 1B).

To analyze the clinicopathological features and the prognoses of the patients according to the miRNA expression, we classified the DLBCL patients into two groups, *i.e.*, those with high *vs.* low miRNA levels relative to controls according to the 2^−ddCt^ values described in Methods. As summarized in Table 1, miR-21 was significantly overexpressed in DLBCLs that presented at an advanced stage (*P* = 0.011), and the miR-17-92 expression was significantly higher in patients with older age (*P* = 0.019) or a poor performance status (PS) (*P* = 0.012). High miR-155 expression was also significantly associated with adverse clinicopathological features, including an older age (*P* = 0.003), an advanced stage (*P* = 0.018), a higher revised-International Prognostic Index (R-IPI) (*P* = 0.031), the presence of B symptoms (*P* = 0.003), a poor PS (*P* = 0.049), and ABC subtype (*P* = 0.043) (Table 1). The higher expression of miR-155 in the ABC subtype than in the GCB subtype was consistent with the results of a previous report [22].

**Table 1:** Correlations between the miRNA expression levels and the clinicopathological variables in patients with DLBCL

Variables^a^		miR-21			miR-17-92 cluster			miR-155		
	All (n = 200)	Low (n = 162)	High (n = 38)	*P*	Low (n = 126)	High (n = 74)	*P*	Low (n = 121)	High (n = 79)	*P*
Age										
Mean (range)	58.1(8-86)	57.56(8-86)	60.58(12-85)	0.271	56.2(8-86)	61.4(12-85)	0.019	55.72(8-86)	61.84(23-85)	0.003
<60	90	76(84.4%)	14(15.6%)	0.261	63(70.0%)	27(30.0%)	0.064	63(70.0%)	27(30.0%)	0.013
≥60	110	86(78.2%)	24(21.8%)		63(57.3%)	47(42.7%)		58(52.7%)	52(47.3%)	
Sex										
Male	113	92(81.4%)	21(18.6%)	0.864	78(69.0%)	35(31.0%)	0.044	71(62.8%)	42(37.2%)	0.442
Female	87	70(80.5%)	17(19.5%)		48(55.2%)	39(44.8%)		50(57.5%)	37(42.5%)	
Primary Site										
Nodal	61	47(77.0%)	14(23.0%)	0.345	39(63.9%)	22(36.1%)	0.856	31(50.8%)	30(49.2%)	0.064
Extranodal	139	115(82.7%)	24(17.8%)		87(62.6%)	52(37.4%)		90(64.7%)	49(35.3%)	
Stage										
1,2	107	93(86.9%)	14(13.1%)	0.011	72(67.3%)	35(32.7%)	0.117	72(67.3%)	35(32.7%)	0.018
3,4	87	63(72.4%)	24(27.6%)		49(56.3%)	38(43.7%)		44(50.6%)	43(49.4%)	
R-IPI group										
Very good + Good	114	96(84.2%)	18(15.8%)	0.120	75(65.8%)	39(34.2%)	0.179	75(65.8%)	39(34.2%)	0.031
Poor	63	47(74.6%)	16(25.4%)		35(55.6%)	28(44.4%)		31(49.2%)	32(50.8%)	
B symptom										
Absent	159	131(82.4%)	28(17.6%)	0.139	98(61.6%)	61(38.4%)	0.652	102(64.2%)	57(35.8%)	0.003
Present	35	25(71.4%)	10(28.6%)		23(65.7%)	12(34.3%)		13(37.1%)	22(62.9%)	
Bulky										
Absent	174	141(81.0%)	33(19.0%)	0.597	107(61.5%)	67(38.5%)	0.374	107(61.5%)	67(38.5%)	0.100
Present	21	16(76.2%)	5(23.8%)		15(71.4%)	6(28.6%)		9(42.9%)	12(57.1%)	
Performance status										
0-1	146	120(82.2%)	26(17.8%)	0.247	98(67.1%)	48(32.9%)	0.012	92(63.0%)	54(37.0%)	0.049
≥2	47	35(74.5%)	12(25.5%)		22(46.8%)	25(53.2%)		22(46.8%)	25(53.2%)	
Serum LDH										
Normal	77	65(84.4%)	12(15.6%)	0.295	48(62.3%)	29(37.7%)	0.983	50(64.9%)	27(35.1%)	0.246
Elevated	96	75(78.1%)	21(21.9%)		60(62.5%)	36(37.5%)		54(56.2%)	42(43.8%)	
Extranodal sites (n)										
0.1	153	126(82.4%)	27(17.6%)	0.163	98(64.1%)	55(35.9%)	0.293	93(60.8%)	60(39.2%)	0.507
≥2	40	29(72.5%)	11(27.5%)		22(55.0%)	18(45.0%)		22(55.0%)	18(45.0%)	
Bone marrow involvement										
Absent	154	125(81.2%)	29(18.8%)	0.546	96(62.3%)	58(37.7%)	0.873	90(58.4%)	64(41.6%)	0.600
Present	25	19(76.0%)	6(24.0%)		16(64.0%)	9(36.0%)		16(64.0%)	9(36.0%)	
Rituximab treatment										
No	91	68(74.7%)	23(25.3%)	0.052	57(62.6%)	34(37.4%)	0.975	55(60.4%)	36(39.6%)	0.843
Yes	105	90(85.7%)	15(14.3%)		66(62.9%)	39(37.1%)		62(59.0%)	43(41.0%)	
GCB *vs.* ABC (Choi)										
GCB	53	45(84.9%)	8(15.1%)	1.000	36(67.9%)	17(32.1%)	0.743	38(71.7%)	15(28.3%)	0.043
ABC	95	79(83.2%)	16(16.8%)		62(65.3%)	33(34.7%)		52(54.7%)	43(45.3%)	

R-IPI, revised-international prognostic index; GCB, germinal center B cell-like; ABC, activated B-cell like

a Several variables contain missing values due to the lack of relevant information in the patients.

### Prognostic implication of miR-21, miR-17-92 and miR-155 expression levels in DLBCL patients

The Kaplan-Meier analysis showed that a high expression of miR-21 and miR-17-92 was significantly associated with decreased overall survival (OS) (*P* = 0.004 and *P* = 0.012, respectively) (Fig. 1C and 1D) and progression-free survival (PFS) (*P* = 0.003 and *P* = 0.014, respectively) (Fig. 1F and 1G) but miR-155 was not (Fig. 1E and H). The multivariate Cox analysis showed that a high miR-21 was an independent predictor for poor survival in the overall patients with DLBCL (for OS, HR = 2.1, *P* = 0.020; for PFS, HR = 2.3, *P* = 0.032) and in those treated with rituximab-combined chemotherapy (for OS, HR = 2.4, *P* = 0.032; for PFS, HR = 2.8, *P* = 0.030) (Table 2). Moreover, a high miR-17-92 was also an independent predictor for poor PFS in overall patients (HR = 2.2; *P* = 0.023) and in those treated with rituximab-combined chemotherapy (HR = 2.6; *P* = 0.030) but not for their OS (data not shown). When the data were evaluated according to the immunophenotypical subgroups of DLBCLs, high miR-21 was significantly correlated with a shorter OS and PFS in the GCB subgroup (for OS, HR = 6.0 and *P* = 0.001; for PFS, HR = 5.4 and *P* = 0.001) but not in the ABC subgroup (Supplementary Table S1 and Fig. S1). Together, these data indicated that miR-21, miR-17-92 and miR-155 are frequently overexpressed in DLBCL tissues and that high levels of miR-21 and miR-17-92 expression are correlated with a poor clinical outcome for the DLBCL patients. In particular, miR-21 was an independent prognostic indicator for both the PFS and OS of patients with DLBCL.

**Table 2:** Prognostic implications of miR-21 expression in DLBCL patients evaluated using the multivariate Cox-regression model

	All patients			Rituximab treated-patients^a^		
	HR	95% CI	*P* value	HR	95% CI	*P* value
Overall survival						
miR-21(high *vs*. low)	2.1	1.1–3.9	0.020	2.4	1.1–5.4	0.032
R-IPI (Poor *vs*. Very good + Good)	4.0	2.2–7.2	<0.001	2.8	1.1–5.4	0.022
Subtype (GCB *vs*. ABC)	1.0	0.5–1.9	0.991	1.2	0.5–2.8	0.606
Progression-free survival						
miR-2 I (high *vs*. low)	2.3	1.1–4.8	0.032	2.8	1.14.9	0.030
R-IPI (Poor *vs*. Very good + Good)	3.0	1.5–6.1	0.002	1.7	0.74.1	0.206
Subtype (GCB *vs*. ABC)	1.1	0.6–2.4	0.717	1.7	0.7–4.3	0.281

R-1P1, revised-international prognostic index; GCB, germinal center B cell-like: ABC, activated B-cell like

a Of the rituximab-treated patients, 85.74 (90/105) were treated using the R-CHOP (rituximab, cyclophosphamide, vincristine, doxorubicin, and prednisone) regimen.

### Forkhead box protein O1 (FOXO1) and phosphatase and tensin homolog (PTEN) are targeted by miR-21 in DLBCLs

Because miR-21 had the strongest prognostic implication, we explored the mechanism by which miR-21 contributes to the pathogenesis of DLBCLs. For this purpose, we focused on FOXO1 and PTEN among the many possible targets of miR-21 that were identified using database and literature searches (*e.g*., www.microrna.org and www.targetscan.org) [17, 23]. PTEN is a tumor suppressor that inhibits the PI3K/AKT pathway and a well-known direct target of miR-21 [23, 24]. FOXO1 is a transcription factor for p27, p21, FasL, and Bim, which function as tumor suppressors by blocking the G_1_/S transition and inducing apoptosis [25-27]. Notably, FOXO1 is also a downstream molecule of the PI3K/AKT pathway. Activated AKT phosphorylates FOXO1, which is subsequently exported from the nucleus into the cytoplasm and degraded by proteasomes [28]. Therefore, we hypothesized that miR-21 might modulate the PI3K/AKT pathway by targeting PTEN upstream and FOXO1 downstream.

**Figure 2:**
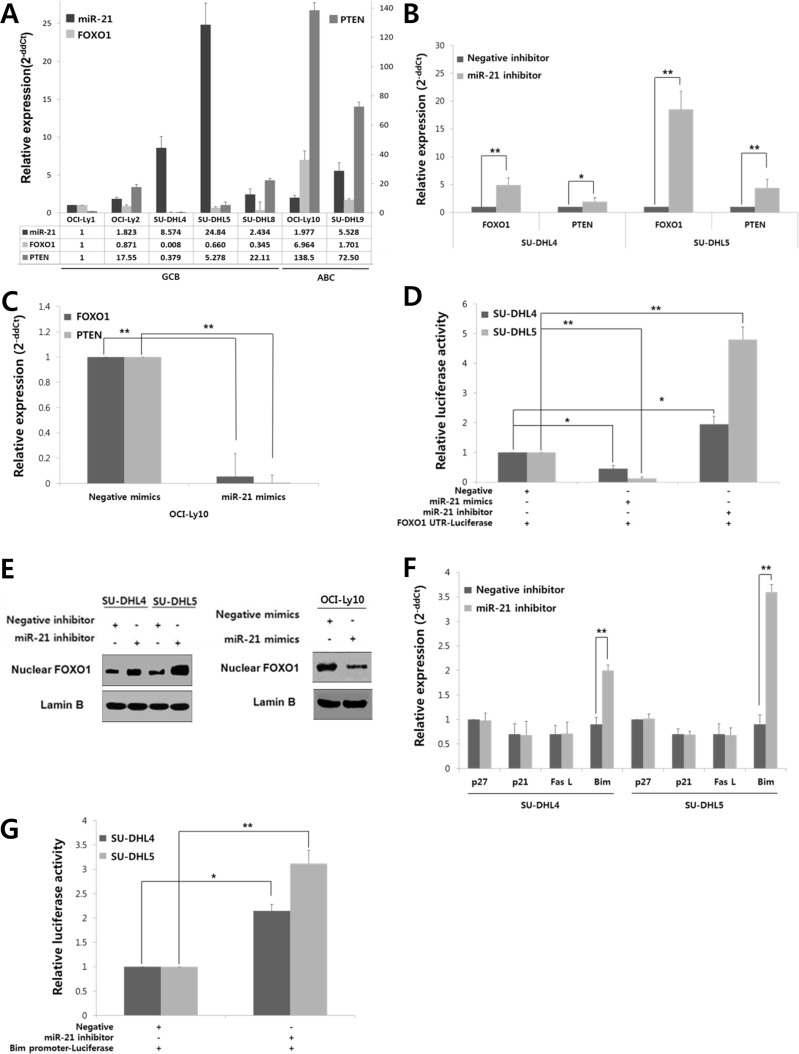
FOXO1, which is directly targeted by miR-21 in addition to PTEN, controls Bim expression at a transcriptional level in DLBCLs **A**, The expression levels of miR-21, FOXO1, and PTEN were evaluated in GCB-DLBCL and ABC-DLBCL cell lines using qRT-PCR. The bar graph shows the relative expression levels (2^−ddCt^) of miR-21, FOXO1, and PTEN in each cell line compared with those of OCI-Ly1 cells. **B**, SU-DHL4 and SU-DHL5 cells were transfected with a miR-21 inhibitor or a negative (scramble) inhibitor using Lipofectamine 2000, and the cells were harvested at 24 hours after transfection. The expression levels of FOXO1 and PTEN in cells that were transfected with the miR-21 inhibitor were compared with those transfected with the negative inhibitor using qRT-PCR. **C**, OCI-Ly10 cells were transfected with miR-21 mimics or negative (scramble) mimics, and the differences in the expression levels of FOXO1 and PTEN between the negative- and miR-21 mimic-cells were assessed using qRT-PCR. **D**, The FOXO1 3′-untranslated region (UTR)-luciferase reporter construct containing many miR-21 binding motifs (5′-AGCUUAU-3′) was co-transfected into SU-DHL-4 and SU-DHL5 cells together with a miR-21 mimic, negative mimic, miR-21 inhibitor or negative inhibitor. At 24 hours post-transfection, the supernatant fractions of the lysed cells were recovered, and the luciferase activities were determined using a luminometer. **E**, At 24 hours after transfection of the miR-21 inhibitor or negative inhibitor into SU-DHL4 and SU-DHL5 cells, or miR-21 mimics or negative mimics into OCI-Ly10 cells, Western blotting was performed to determine FOXO1 level in the nucleus of tumor cells. **F**, Changes in the expression of FOXO1 target genes, including p27, p21, FasL and Bim, were evaluated using qRT-PCR after inhibiting the expression of miR-21 in SU-DHL4 and SU-DHL5 cells. **G**, A Bim promoter luciferase-reporter construct containing many copies of the FOXO1 binding motif (5′-GTAAACAA-3′) was co-transfected with either a miR-21 inhibitor or negative inhibitor into SU-DHL4 or SU-DHL5 cells. At 24 hours post-transfection, the supernatant fractions of the lysed cells were recovered, and the luciferase activities were measured using a luminometer. The values presented in the histogram are the mean values ± SD. Statistically significant differences are indicated by * and **, which signify *P* < 0.05 and *P* < 0.005, respectively, as determined using the paired t-test.

To address this hypothesis, we first screened the expression levels of miR-21, FOXO1 and PTEN in a variety of DLBCL cell lines. As shown in Fig. 2A, the miR-21 expression level was inversely correlated with both the FOXO1 and PTEN levels in DLBCL cells. Furthermore, PTEN expression was low in the GCB-DLBCL-derived cell lines compared to the ABC-DLBCL-derived cell lines (Fig. 2A). This expression pattern of PTEN in DLBCL cells was consistent with a previous report [7]. For further study, SU-DHL4 and SU-DHL5 cells, which have high miR-21/low FOXO1/low PTEN expression, and OCI-Ly10 cells, which have low miR-21/high FOXO1/high PTEN expression, were selected. The expression of FOXO1 and PTEN were significantly increased in SU-DHL4 and SU-DHL5 by transfection with a miR-21 inhibitor (Fig. 2B). Consistently, transfection with miR-21 mimics resulted in the significant down-regulation of PTEN and FOXO1 in OCI-Ly10 (Fig. 2C). Furthermore, transfection with miR-21 mimics led to decreased FOXO1 mRNA 3′-UTR luciferase activity, but miR-21 inhibitor increased its luciferase activity (Fig. 2D). Considering the function of FOXO1 as transcription factor we evaluated the alteration of nuclear FOXO1 level by miR-21. As shown in Fig. 2E, nuclear FOXO1 was increased in SU-DHL4 and SU-DHL5 by miR-21 inhibitor but decreased in OCI-Ly10 cells by miR-21 mimics.

In human DLBCL tissues, FOXO1 expression was observed by immunohistochemistry (IHC) in 80.8% (126/156) of the cases with variable intensities and staining patterns, which were related to the miR-21 level. Briefly, cases with a low miR-21 showed increased nuclear expression of FOXO1, whereas those with a high miR-21 frequently exhibited cytoplasmic expression (Supplementary Fig. S2A). For the statistical analysis, the FOXO1-IHC score was calculated as the nuclear intensity minus the cytoplasmic intensity. Accordingly, the patients were divided into two groups, *i.e.*, those with predominant nuclear staining (a FOXO1-IHC score of ≥ 0) and those with predominant cytoplasmic staining (a score of < 0). Nuclear predominant FOXO1 was significantly correlated with a low miR-21in the DLBCL tissues (*P* = 0.0002) (Supplementary Fig. S2B).

Taken together, these data indicate that miR-21 suppresses FOXO1 and PTEN expression in DLBCL and that FOXO1 is a direct target of miR-21 in DLBCL cells.

### MiR-21-regulated FOXO1 controls Bim expression at a transcriptional level

To determine the function of FOXO1 regulated by miR-21 in DLBCL, we investigated the changes of FOXO1 transcriptional target molecules, including p27, p21, FasL and Bim. Notably, transfection of SU-DHL4 and SU-DHL5 cells with the miR-21 inhibitor resulted in the up-regulation of Bim expression but had little effect on the others (Fig. 2F). Moreover, miR-21 inhibitor increased the luciferase activity of vectors containing the 5′-FOXO1-binding sites of the Bim promoter region in these cells, which suggested that Bim expression is increased at the transcriptional level by FOXO1 (Fig. 2G). Consistently, treatment with a transcriptional inhibitor (actinomycin D) blocked the induction of Bim even after the miR-21 inhibitor transfection (Supplementary Fig. S3).

In human DLBCL tissues, Bim expression showed a tendency of inverse relationship with the miR-21 expression. Briefly, Bim mRNA level was lower in high miR-21 group compared to low miR-21 group (Supplementary Fig. S4A). Bim expression by IHC was categorized into low (none to mild) *vs*. high (moderate to strong) according the staining intensity. Among cases with high miR-21, low Bim expression was more commonly observed rather than high Bim expression. In contrast, high Bim expression was more frequent in those with low miR-21 although the difference was statistically insignificant (Supplementary Fig. S4B).

Taken together, these data indicated that FOXO1 up-regulates pro-apoptotic Bim at the transcriptional level and thus FOXO1/Bim axis would be an important pathway that is regulated by miR-21 in DLBCLs.

### MiR-21 activates the PI3K/AKT/mTOR pathway and FOXO1 inactivates mTOR in DLBCL

**Figure 3:**
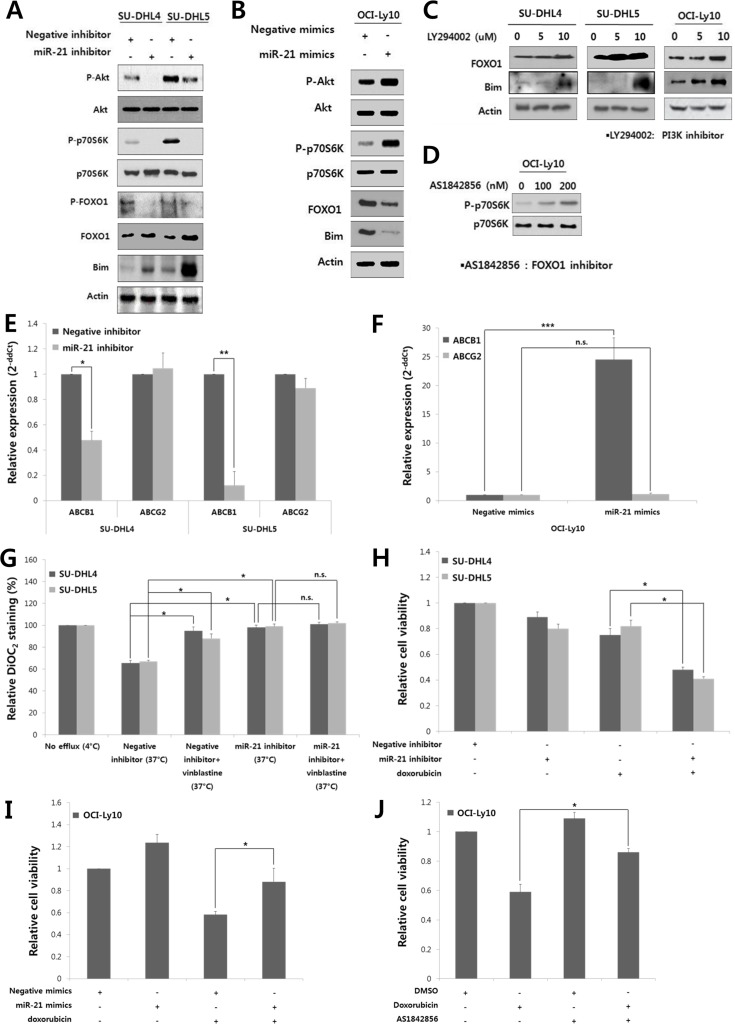
MiR-21 regulates the PI3K/AKT/mTOR/FOXO1 pathway and involved in the drug resistance and proliferation of DLBCL cells At 24 hours after transfection of **A**, the miR-21 inhibitor or negative inhibitor into SU-DHL4 and SU-DHL5 cells or **B**, miR-21 mimics or negative mimics into OCI-Ly10 cells, Western blotting was performed to determine the levels of phospho-AKT, AKT, phospho-p70S6K, p70S6K, phospho-FOXO1, FOXO1 and Bim. **C**, SU-DHL4, SU-DHL5, and OCI-Ly10 cells were treated with increasing doses of LY294002, a PI3K inhibitor. At 24 hours after incubation, Western blotting was performed to determine the levels of FOXO1 and Bim. **D**, OCI-Ly10 cells were treated with increasing doses of AS1842856, a functional inhibitor of FOXO1, and 2 hours after incubation, Western blotting was performed to determine the levels of phospho-p70S6K and p70S6K. At 24 hours after transfection of **E**, the miR-21 inhibitor or negative inhibitor into SU-DHL4 and SU-DHL5 cells, or **F**, miR-21 mimics or negative mimics into OCI-Ly10 cells, the expression levels of ABCB1 (MDR1) and ABCG2 were evaluated using qRT-PCR. **G**, SU-DHL4 and SU-DHL5 cells were treated with the miR-21 inhibitor or negative inhibitor for 24 hours and co-incubated with the efflux-blocking drug, vinblastine, for the last 30 minutes. The cells were then stained with DiOC_2_, and their drug efflux activity was analyzed. A decrease in the percentage of DiOC_2_-staining cells determined using FACS represents an increase in the population of cells with drug efflux activity. **H**, The effect of miR-21 inhibition on the doxorubicin-induced cytotoxicity in SU-DHL4 and SU-DHL5 cells was evaluated using the CCK8 assay. **I**, The effect of miR-21 overexpression on the doxorubicin-induced cytotoxicity in OCI-Ly10 cells was assessed using the CCK8 assay. **J**, The relative rates of cell proliferation of OCI-Ly10 cells treated with DMSO (control) and doxorubicin and/or the FOXO1 inhibitor (AS1842856) were determined using the CCK8 assay. The values presented in the histogram are the mean values ± SD. Statistically significant differences are indicated by *, ** and ***, which signify *P* < 0.05, *P* < 0.005 and *P* < 0.0005, respectively, as determined using the paired *t*-test.

Given that the expression of FOXO1 and PTEN was down-regulated by miR-21 in DLBCLs (Fig. 2B and C), we further investigated if miR-21 activated the PI3K/AKT/mTOR pathway. MiR-21 inhibitor suppressed AKT and mTOR activity as shown by decrease in phospho-AKT and phospho-70S6K in SU-DHL4 and SU-DHL5 cells (Fig. 3A). At the same time, the phospho-FOXO1 level was diminished, but the amount of total FOXO1 was increased along with Bim up-regulation (Fig. 3A). In contrast, miR-21 mimics activated the PI3K/AKT/mTOR pathway in OCI-Ly10 cells but down-regulated FOXO1 and Bim (Fig. 3B). These data suggested that miR-21 activates the PI3K/AKT/mTOR pathway in DLBCL. Accordingly, miR-21 inhibition rescued FOXO1 from AKT activity-mediated phosphorylation and putatively subsequent degradation. Consistently, a functional inhibitor of PI3K, LY294002, up-regulated the expression of FOXO1 and Bim in DLBCL cells (Fig. 3C), which further suggested that the FOXO1/Bim axis may be an important target regulated by the PI3K/AKT signaling in DLBCL.

To further see the role of FOXO1 in PI3K/AKT/mTOR pathway of DLBCL, we examined if FOXO1 had an inhibitory effect on mTOR activity as previously reported in other types of cells [29, 30]. A functional inhibitor of FOXO1, AS1842856, activated mTOR pathway as shown by increased phospho-p70S6K in OCI-Ly10 cells (Fig. 3D).

Taken together, these results indicated that miR-21 suppresses FOXO1 and its transcriptional target Bim directly by binding to the 3′-UTR of FOXO1 and indirectly by activating the PI3K/AKT pathway in DLBCL.

### MiR-21 up-regulates the expression and activity of multidrug resistant protein 1 (MDR1) to induce drug resistance in DLBCL cells

Finally, we evaluated the effect of miR-21 on cell viability and drug resistance in DLBCL. MDR1 (encoded by the *ABCB1* gene) functions as drug efflux pump with broad substrate specificity [31], and is known to be up-regulated by AKT activity [32]. Thus, we examined if miR-21 affected MDR1 expression in DLBCL cells. The miR-21 inhibitor transfection in SU-DHL4 and SU-DHL5 cells significantly suppressed the MDR1 expression, but with little effect on another transport-related protein, ABCG2 (Fig. 3E). In contrast, miR-21 mimics up-regulated the expression of MDR1 but not of ABCG2 in OCI-Ly10 cells (Fig. 3F). DiOC_2_ was effluxed from SU-DHL4 and SU-DHL5 cells by up to 40% at the basal level (Fig. 3G, “negative inhibitor (37°C)”), which was blocked by vinblastine (an efflux-blocking agent). Notably, the drug efflux activity was significantly blocked by the miR-21 inhibitor (Fig. 3G).

In addition, the miR-21 inhibitor reduced the proliferation of SU-DHL4 and SU-DHL5 cells (Supplementary Fig. S5). Combined treatment with the miR-21 inhibitor and doxorubicin synergistically suppressed the proliferation and viability of these cells (Fig. 3H). In contrast, treatment with miR-21 mimics increased the proliferation of OCI-Ly10 cells and antagonized the cytotoxic effect of doxorubicin (Fig. 3I). Interestingly, the cytotoxic effect of doxorubicin was also antagonized by a FOXO1 inhibitor (AS1842856) in OCI-Ly10 cells (Fig. 3J). These data indicated that miR-21 and FOXO1 may be involved in the drug resistance of DLBCL cells and that miR-21 inhibition can sensitize these cells to doxorubicin.

**Figure 4:**
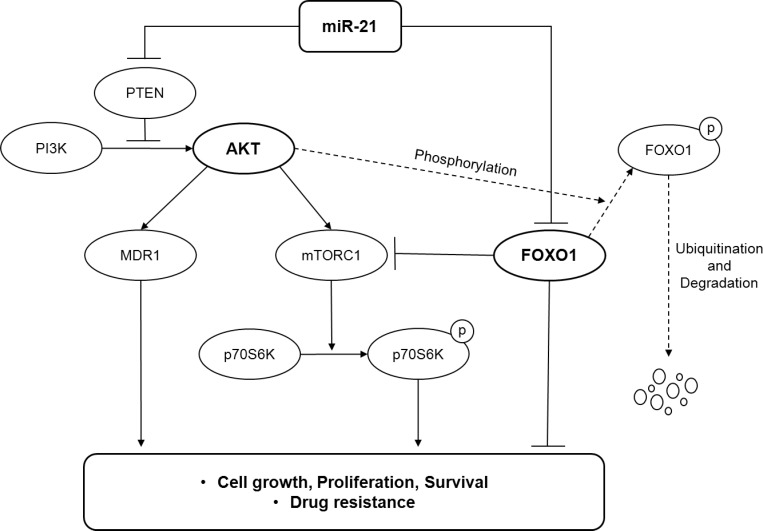
A model illustrating the regulation of the PI3K/AKT/mTOR/FOXO1 pathway by miR-21 at multiple levels in DLBCL

Considering all of the results in this study, we propose a model in which miR-21 plays an oncogenic role by activating the PI3K/AKT/mTOR pathway and suppressing the FOXO1/Bim pathway at multiple levels in DLBCL (Fig. 4).

## DISCUSSION

Although miR-21, miR-17-92 and miR-155 are oncogenic miRNAs reported to be overexpressed in DLBCL [10, 23], comprehensive analysis of these miRNAs in a large cohort of DLBCL patients was rare, and their prognostic implications were conflicting depending on the types of patients-derived samples and study cohort [33, 34]. In the present study, the expressions of the miR-21, miR-17-92 and miR-155 were significantly up-regulated in DLBCL tissues, and their levels showed a strong positive correlation with each other. To avoid the confounding effect of the treatment regimen, we investigated the prognostic implications of miRNAs in patients with rituximab treatment and in overall patients, respectively, because patients who did not treated with rituximab were received variable treatment regimens such as CHOP, COPBLAM (cyclophosphamide, vincristine, prednisone, bleomycin, doxorubicin and procarbazine) and HDMTX (high dose methotrexate). Overexpression of these miRNAs was associated with adverse clinicopathological features, and high miR-21 and miR-17-92 were independent poor prognostic factors in overall DLBCL patients and those treated with rituximab. These data suggested that miR-21, miR-17-92 and miR-155 might cooperatively contribute to the pathogenesis of DLBCL and that they are important constituents forming an oncogenic miRNA signature of DLBCLs with a poor clinical outcome. Although we determined the levels of the premature miRNAs using formalin-fixed paraffin-embedded (FFPE) tissues, it has been reported that the expression levels of premature miRNAs are well correlated with those of mature miRNAs [35, 36]. Considering that the miRNA levels in tumor tissues and serum show strong positive correlations [34], the serum levels of the miRNAs analyzed in this study could be utilized as prognostic biomarkers.

This study demonstrated that FOXO1 is a novel direct target of miR-21 and is implicated in the pathogenesis of DLBCL [27]. FOXO1 is also important for the development and differentiation of B-cell [37], and the inactivation of FOXO1 is required for optimal B-cell proliferation [38]. Regarding the lymphoid malignancies, FOXO1 was shown to be a tumor suppressor in Hodgkin lymphoma [39]. Dysregulation of FOXO1 by an inactivating mutation has been recently reported in DLBCLs [40]. However the functional role of FOXO1 in the pathogenesis of DLBCL remains unknown. In our study, the inhibition of FOXO1 resulted in resistance to apoptotic stimuli (*i.e.*, doxorubicin), thereby suggesting a pro-apoptotic role for FOXO1 in DLBCL cells. Moreover, the pro-apoptotic Bim was up-regulated by FOXO1 following the inhibition of miR-21, but other FOXO1 target molecules, including p27, p21, and FasL, were not affected. FOXOs interact with various transcriptional cofactors, which are suspected to specify the target genes of FOXO1 [26]. For example, RUNX3 in cooperation with FOXO up-regulates Bim in gastric cancer cells [41]. Therefore, it is possible that cofactors may contribute to the selective regulation of the FOXO1/Bim axis by miR-21 in the DLBCL cells. Together, the results of the present study demonstrate that FOXO1 may function as a tumor suppressor in DLBCLs.

Despite mounting evidence of a tumor suppressive role for FOXO1, the mechanism by which FOXO1 activity is regulated remains unclear. It has been known that FOXO1 activity is generally regulated by posttranscriptional modification rather than genetic aberration [42, 43]. Deletion of genetic loci encompassing FOXO1 was observed in only a portion of Hodgkin lymphomas, and a FOXO1 mutation accounted for 8.6% of DLBCL cases [39, 40]. Moreover, the level of FOXO1 expression was not dependent on the FOXO1 mutation in DLBCLs [40], which suggests that other factors might be involved in the regulation of FOXO1 expression in DLBCLs. Other mechanisms reported to be important for the regulation of FOXO1 are PI3K/AKT signaling and miRNA [39, 42]. Exclusion of FOXO1 from the nucleus and thus its presence in the cytoplasm was been observed in DLBCLs showing chronic active BCR signaling or PI3K/AKT activation [44]. Among the miRNAs, miR-27a, miR-96, miR-182, and miR-183 suppressed FOXO1 expression in breast cancer, melanoma, and Hodgkin lymphoma [39, 42]. However, the association between miR-21 and FOXO1 expression has never been addressed. This study demonstrated that miR-21 down-regulated FOXO1 in both direct and indirect manners by binding the 3′-UTR of FOXO1 and by activating the PI3K/AKT pathway in DLBCLs.

The PI3K/AKT pathway is regarded to be an important oncogenic signaling pathway in both the GCB and ABC types of DLBCL [5, 7]. Activation of the PI3K signaling blocks apoptosis and is required for constitutive NF-κB activation in ABC-DLBCL [45]. However, the loss of PTEN occurs in only 14% of non-GCB DLBCLs, and ABC-DLBCL cells often show AKT activation even in the presence of PTEN [7]. Therefore, activation of the PI3K/AKT pathway in ABC-DLBCLs has been suspected to be independent of PTEN. In contrast, the loss of PTEN occurs in 55% of GCB-DLBCLs, and AKT activation in GCB-DLBCL cells is frequently associated with the PTEN loss, which makes tumor cells addicted to oncogenic PI3K signaling [7]. However, the mechanism of PTEN downregulation in DLBCLs remains to be elucidated. In this study, we demonstrated that miR-21 activated the PI3K/AKT/mTOR pathway by targeting PTEN at the upstream level and by targeting the FOXO1/Bim pathway at the downstream level as schematically illustrated in Fig. 4. Furthermore, the present study showed that FOXO1 activity may counteractively inhibit the mTORC1 pathway in DLBCL cells. Moreover, miR-21 had a prognostic significance for DLBCL patients with the GCB phenotype but not the ABC phenotype. Thus, we suggest that miR-21 might be involved in the oncogenic addiction of GCB-DLBCLs to the PI3K/AKT pathway.

In this study, miR-21 inhibition suppressed DLBCL cell proliferation and sensitized the DLBCL cells to doxorubicin. These data were consistent with those of a previous report showing that miR-21 expression was correlated with chemoresistance in DLBCL cells, which was mediated by PTEN suppression [17]. Here, we further showed that miR-21-mediated chemoresistance involved the up-regulation of MDR1 expression and activity. In addition, considering that the level of Bim predicts the sensitivity of DLBCLs to chemotherapeutic agents [46], miR-21 might further contribute to chemoresistance by down-regulating the activity of the FOXO1/Bim pathway. Taken together, this study demonstrated that miR-21 may play an oncogenic role in DLBCL by comprehensively regulating the PI3K/AKT/mTOR/FOXO1 pathway (Fig. 4) and that a high miR-21 is associated with a poor prognosis for DLBCL patients. Thus, targeting miR-21 would have therapeutic relevance for the management of DLBCLs. However, further studies using *in vivo* model would be needed to provide more solid evidence for the oncogenic role of miR-21 in DLBCL.

Moreover, according to previous studies, both miR-17-92 and miR-155 also regulate the PI3K/AKT pathway. MiR-19 is a key oncogenic component of the miR-17-92 cluster, and it suppresses PTEN expression, consistently activates the AKT/mTOR pathway and promotes Myc-induced lymphomagenesis [47]. MiR-155 activates the PI3K/AKT pathway through directly targeting p85α, a negative regulatory subunit of PI3K, in DLBCL [48]. Thus, it is possible that miR-21, miR-17-92, and miR-155 cooperatively contribute to the activation of the PI3K/AKT pathway in DLBCLs.

In summary, we showed that high miR-21 and miR-17-92 expression had poor prognostic implications in patients with DLBCL. FOXO1 was revealed to be a novel direct target of miR-21. This study demonstrates that miR-21 may play an important oncogenic role in DLBCL through activating the PI3K/AKT/mTOR pathway at multiple levels by targeting PTEN and by suppressing the FOXO1/Bim pathway, thus being a potential therapeutic target for DLBCL.

## MATERIALS AND METHODS

### Patients and samples

A total of 200 patients with DLBCL were enrolled in this study. All patients were diagnosed with DLBCL from 1997 to 2010 at Seoul National University Hospital (SNUH) according to the current World Health Organization criteria, and FFPE tumor tissues appropriate for miRNA analysis were available. The clinical data were retrieved from the medial records by hemato-oncologists (T.M.K. and D.S.H.). This study was performed in accordance with the Declaration of Helsinki and approved by the Institutional Review Board (IRB) of SNUH (H-1012-053-344).

### Cell lines and reagents

Cell lines derived from GCB-DLBCLs (*i.e.*, OCI-Ly1, OCI-Ly2, SU-DHL4, SU-DHL5, and SU-DHL8) and ABC-DLBCLs (*i.e.*, OCI-Ly10 and SU-DHL9) were kindly provided by Prof. Megan S. Lim (University of Michigan, Ann Arbor, MI, USA). All of the cell lines were cultured and subtypes in terms of the cell-of-origin according to the instructions of the American Type Culture Collection or the German Collection of Microorganisms and Cell Cultures. Actinomycin D, LY294002, AS1842856, vinblastine and doxorubicin were all purchased from Sigma (St. Louis, MO, USA).

### IHC

FFPE tissues were subjected to IHC using primary antibodies as follows: CD10 (56C6, Novocastra, Newcastle Upon Tyne, UK), BCL-6 (LN22, Novocastra), MUM1 (MUM1P, Dako, Glostrup, Denmark), GCET1 (polyclonal, Abcam, Cambridge, UK), FOXP1 (polyclonal, Abcam), FOXO1 (C29H4, Cell Signaling, Danvers, MA, USA) and Bim (Y36, Abcam) using the Ventana Benchmark XT automated staining system (Ventana Medical Systems, Tucson, AZ, USA). DLBCL cases were immunohistochemically sub-grouped into GCB or ABC phenotypes according to the Choi algorithms, as previously described [49]. FOXO1 expression was evaluated according to the staining intensity (0-3) and location (nuclear vs. cytoplasmic).

### qRT-PCR for miRNAs and mRNAs

Total RNA was extracted from the FFPE tissues using a RecoverAll^TM^ Total Nucleic Acid Isolation for FFPE kit (Applied Biosystems, Foster City, CA, USA) following the manufacturer's protocol. The levels of the premature forms of miR-21, the miR-17-92 and miR-155 were determined by qRT-PCR using a one-step SYBRPrimeScript RT-PCR kit (Takara, Seto, Japan) with U6 snRNA (#4373381, Applied Biosystems) as an internal control. The expression levels of the miRNAs were compared with those of 11 normal (i.e., non-neoplastic) tonsils used as controls. The relative expression level (2-ddCt) of each miRNA was calculated as follows: dCt = Ct_(miRNA)_ - Ct_(U6);_ ddCt = dCt_(case)_ - mean dCt_(control)_.

Total RNA was extracted from DLBCL cell lines using TRIzol (Life Technologies, Carlsbad, CA, USA). The miR-21 level was evaluated by qRT-PCR using Mir-X miRNA first strand synthesis and SYBR^®^ qRT-PCR kits (Clontech Laboratories, Mountain View, CA, USA) with U6 snRNA as an internal control. The expression of p27, p21, FasL, Bim, MDR1, and ABCG2 mRNA in DLBCL cells was evaluated by qRT-PCR using a PrimeScript^TM^ 1^st^ strand cDNA synthesis kit (Takara Bio, Otsu, Shiga, Japan) with GAPDH as an internal control. All PCR reactions were performed in triplicate using a Step One Plus thermocycler (Applied Biosystems).

The sequences of the primers used for qRT-PCR for miRNA and mRNA are summarized in Supplementary Table S2.

### Transfection of miRNA inhibitors or mimics

The cells were placed in six-well plates (5 × 10^5^ cells per well) in opti-MEM media (Qiagen, Duesseldorf, Germany) and were transfected with either miR-21 inhibitors or mimics (sequences shown in Supplementary Table S3) using Lipofectamine 2000 (Invitrogen, Carlsbad, CA, USA). The transfected cells were cultured for 6 hours, and the culture medium was then replaced with fresh complete medium. The cells were harvested at 24 hours after transfection.

### Cell viability assay

The cell viability was monitored using the Cell Counting Kit-8 (CCK8) (Dojindo Molecular Technologies, Kumamoto, Japan) according to the manufacturer's protocol. All of the experiments were repeated at least three times.

### Reporter gene assay

The pLenti-III-UbC-Luc-FOXO1 3′-untranslated region (UTR) luciferase-reporter construct (Applied Biological Materials Inc., Richmond, BC, Canada) containing many copies of the miR-21 binding motif (5′-AGCUUAU-3′) was co-transfected with miR-21 mimics/inhibitors or negative (*i.e.*, scramble) mimics/inhibitors into DLBCL cells using Lipofectamine 2000 (Supplementary Fig. S6A). The Bim promoter luciferase-reporter construct (pGL2 vector) (Promega, Madison, WI, USA) containing many copies of the FOXO1 binding motif (5′-GTAAACAA-3′) was co-transfected with a miR-21 inhibitor or negative inhibitor into DLBCL cells (Supplementary Fig. S6B). At 24 hours post-transfection, the cells were lysed, and the supernatant fractions were recovered and the luciferase activities were determined using a luminometer (FB12 luminometer; Berthold Detection Systems, Pforzheim, Germany).

### Western blotting

The total cellular protein was extracted using lysis buffer. To obtain cytoplasmic and nuclear extracts, the cells were suspended in ice-cold hypotonic buffer and centrifuged. The supernatant contained the cytoplasmic fraction, and the nuclear fraction was extracted from the remnant pellet using high-salt buffer. A total of 10-50 μg of protein was electrophoresed in SDS-PAGE gels and transferred to polyvinylidene difluoride membranes (Millipore, Bedford, MA, USA). The membranes were then incubated with the following antibodies: anti-phospho-AKT, anti-AKT, anti-phospho-p70S6K, and anti-p70S6K antibodies (Cell Signaling); and anti-phospho-FOXO1, anti-FOXO1, anti-Bim, anti-β-actin, and anti-lamin B antibodies (Santa Cruz biotechnology, Dallas, TX, USA). The immunoblots were visualized using an enhanced chemiluminescence detection system (Amersham Pharmacia Biotech, Uppsala, Sweden).

### Drug efflux assay

Drug efflux activity was assessed using a multidrug resistance direct dye efflux kit (Millipore, Temecula, CA, USA) according to the manufacturer's instructions. A decrease in the percentage of DiOC_2_-staining cells determined using FACS Canto II (BD Bioscience, San Jose, CA, USA) was considered indicative of drug efflux activity.

### Statistical analysis

The statistical analyses were performed using SPSS software (version 21; IBM Corporation, Armonk, NY, USA) and R 3.0.2 (R Development Core Team, Vienna, Austria). The cut-off values for high vs. low expression of each miRNA relative to that of the normal controls (2^−ddCt^) were selected based on receiver operating characteristic (ROC) curve analysis, and thereby determined to be 7.9656 for miR-21, 7.5795 for miR-17-92, and 23.1139 for miR-155. The relationships or correlations between the groups were assessed using the χ^2^ test, Fisher's exact test, Student's t-test, or Pearson's correlation analysis. Kaplan-Meier method with the log-rank test and Cox proportional hazards regression analysis were used for the survival analyses. All statistical tests were two-sided, and statistical significance was defined as *P* < 0.05.

## SUPPLEMENTARY MATERIAL, TABLES AND FIGURES


