# Supplementary material for: MicroRNA-21 plays an oncogenic role by targeting FOXO1 and activating the PI3K/AKT pathway in diffuse large B-cell lymphoma
Source: Oncotarget. 2015 Mar 30;6(17):15035–49. doi: 10.18632/oncotarget.3729 (PMC4558134; doi:10.18632/oncotarget.3729)
Supplement: Supplementary file 1 [file oncotarget-06-15035-s001.pdf]

MicroRNA-21 plays an oncogenic role by targeting FOXO1 and activating the PI3K/AKT pathway in diffuse large B-cell lymphoma

Supplementary Material

Supplementary Table S1. Prognostic implication of the miR-21 expression analyzed by multivariate Cox-regression model in DLBCL patients with GCB and ABC subtype.

		GCB-like			ABC-like		
		HR	95% CI	*p*	HR	95% CI	*p*
Overall survival							
miR-21	high vs. low	5.957	2.105-16-859	0.001	1.215	0.522-2.830	0.651
R-IPI	poor vs. very good + good	4.027	1.355-11.970	0.012	4.578	2.167-9.669	<0.001
Progression free survival							
miR-21	high vs. low	5.376	1.592-18.161	0.007	1.221	0.445-3.348	0.698
R-IPI	poor vs. very good + good	2.078	0.530-8.152	0.294	4.457	1.886-10.553	0.001

GCB-like and ABC-like subtype were classified using Choi classifier.

Supplementary Table S2. List and sequences of primers used for qRT-PCR .

Name		Sequence
miR-21	forward	5'-TGTCGGGTAGCTTATCAGACT-3'
	reverse	5'-TGTCAGACAGCCCATCGACT-3'
miR-17-92 cluster	forward	5'-GTCAGAATAATGTCAAAGTGCTT-3'
	reverse	5'-TCACCATAATGCTACAAGTGCCT-3'
miR-155	forward	5'-CTGTTAATGCTAATCGTGATAGG-3'
	reverse	5'-CTGTTAATGCTAATATGTAGGAG-3'
miR-21[a]	forward	5'-TAGCTTATCAGACTGATGTTGA-3'.
FOXO1	forward	5'-GCCATGTAAGTCCCATCAGGA-3'
	reverse	5'-ATCGGAACAAGAACGTGGAATC-3'
PTEN	forward	5′-CCAGGACCAGAGGAAACCT-3′
	reverse	5′-GCTAGCCTCTGGATTTGA-3′
p21	forward	5'-AGGAGGCCCGTGAGCGATGGAAC-3'
	reverse	5'-ACAAGTGGGGAGGAGGAAGTAGC-3'
p27	forward	5'-GGTTAGCGGAGCAATGCG-3'
	reverse	5'-TCCACAGAACCGGCATTTG-3'
FasL	forward	5'-GCCCTTCAATTACCCATATCCC-3'
	reverse	5'-GGCAACCAGAACCATGAAAAACA-3'
Bim	forward	5'-TGGCAAAGCAACCTTCTGATG-3'
	reverse	5'-GCAGGCTGCAATTGTCTACCT-3'
ABCB1/MDR1	forward	5'-GCCTGGCAGCTGGAAGACAAATAC-3'
	reverse	5'-GCAGGCTGCAATTGTCTACCT-3'
ABCG2	forward	5'-CCGCGACAGTTTCCAATGACCT-3'
	reverse	5'-GCCGAAGAGCTGCTGAGAACTGTA-3'

[a]for DLBCL cell line

Supplementary Table S3. Sequences of miRNA mimics and inhibitors.

Mimics/inhibitors		Sequence
miR-21 mimics	sense	5’-UAGCUUAUCAGACUGAUGUUGA-3’
	antisense	5’-UCAACAUCAGUCUGAUAAGCUA-3’
Negative mimics	sense	5’-UUGUACUACACAAAAGUACUGUU-3’
	antisense	5’-CAGUACUUUUGUGUAGUACAAUU-3’
miR-21 inhibitor		UCAACAUCA GUCUGAUAAGCUA
Negative inhibitor		CAGUACUUUUGUGUAGUACAA

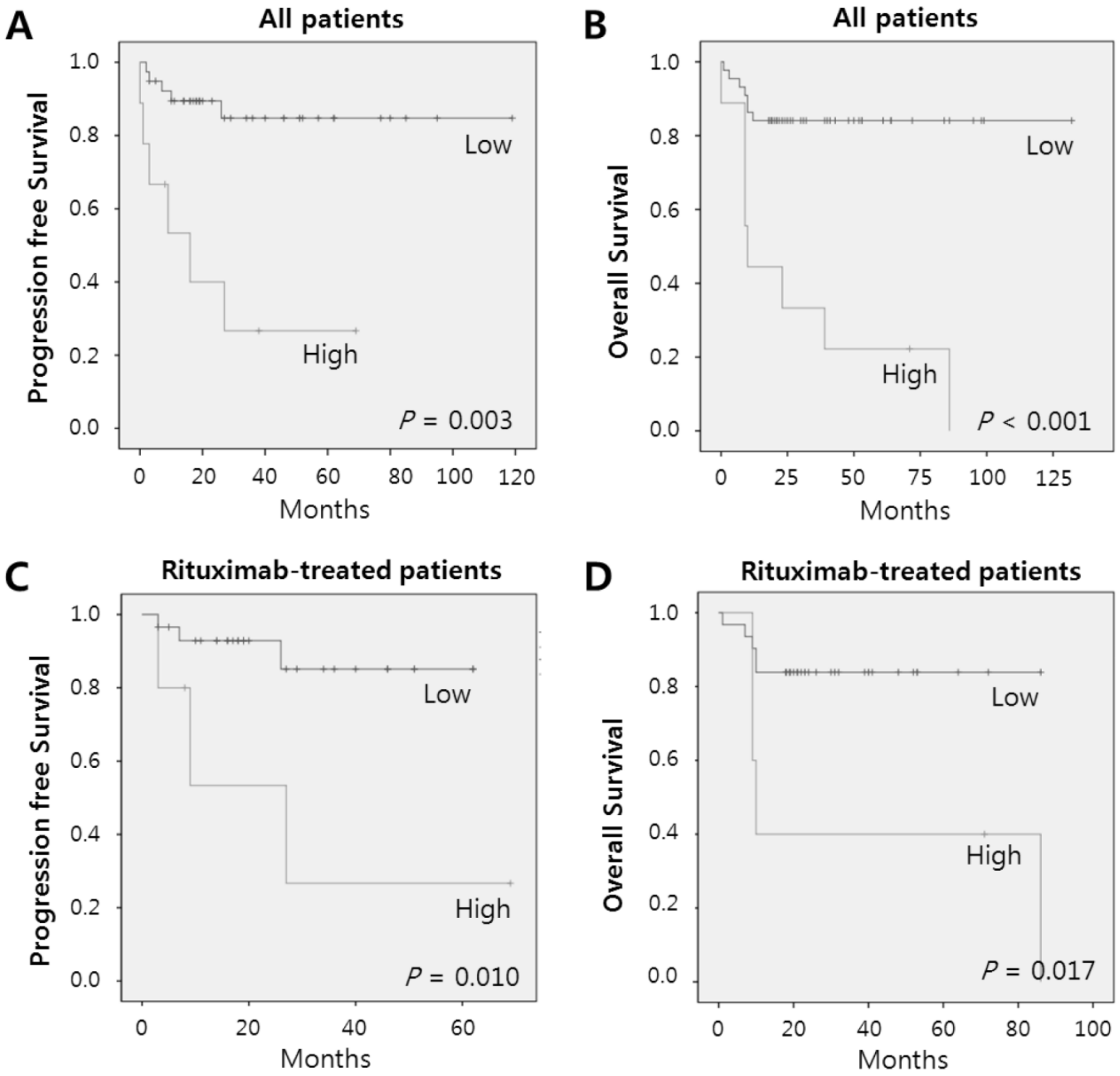

Supplementary Figure S1. Kaplan-Meier survival curves with the log-rank test according to the miR-21 levels in GCB DLBCL patients by Choi classifier.

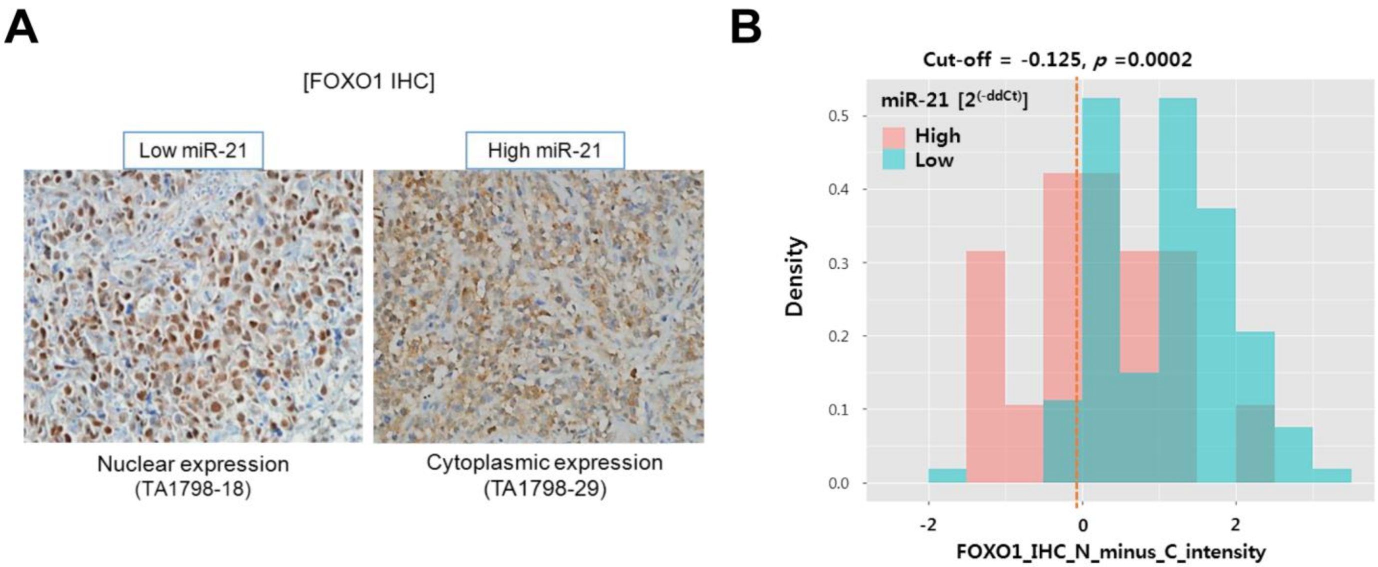

Supplementary Figure S2 A, Representative FOXO1 immunohistochemistry (IHC) images of DLBCL tumors with low *vs.* high expression levels of miR-21. B, The FOXO1 IHC-based expression scores were calculated as the nuclear intensity minus the cytoplasmic intensity of FOXO1 staining considering that FOXO1 functions in the nucleus. Accordingly, the DLBCL cases were divided into two groups as follows: nuclear (*i.e.*, high FOXO1 IHC score) and cytoplasmic (*i.e.*, low FOXO1 IHC score) predominant staining groups. The relationship between the FOXO1-IHC score and the levels of miR-21 expression was analyzed using the Fisher’s exact test.

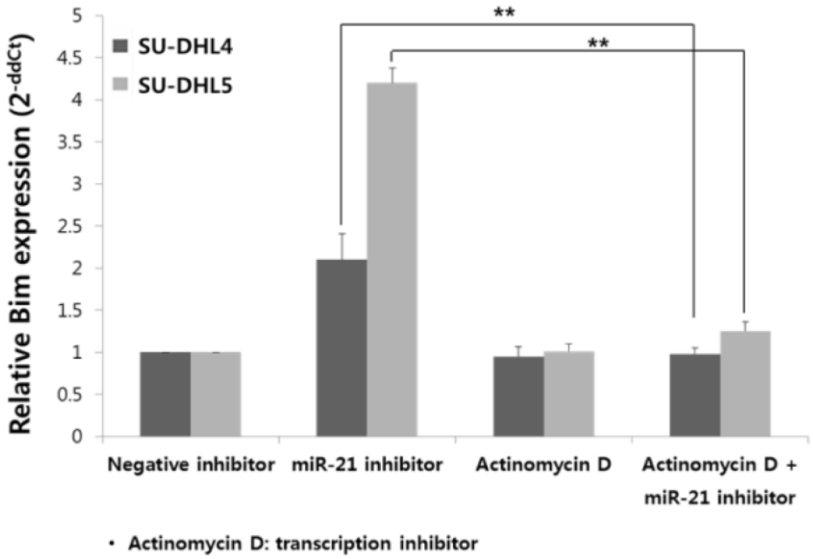

Supplementary Figure S3. SU-DHL4 and SU-DHL5 cells were transfected with either a miR-21 inhibitor or a negative inhibitor followed by treatment with the transcription inhibitor, actinomycin D. At 24 hours after transfection, the level of Bim expression was evaluated using qRT-PCR. The values presented in the histogram are the mean values ± SD. Statistically significant differences are indicated by **, which signify $P < 0.005$ as determined using the paired t-test.

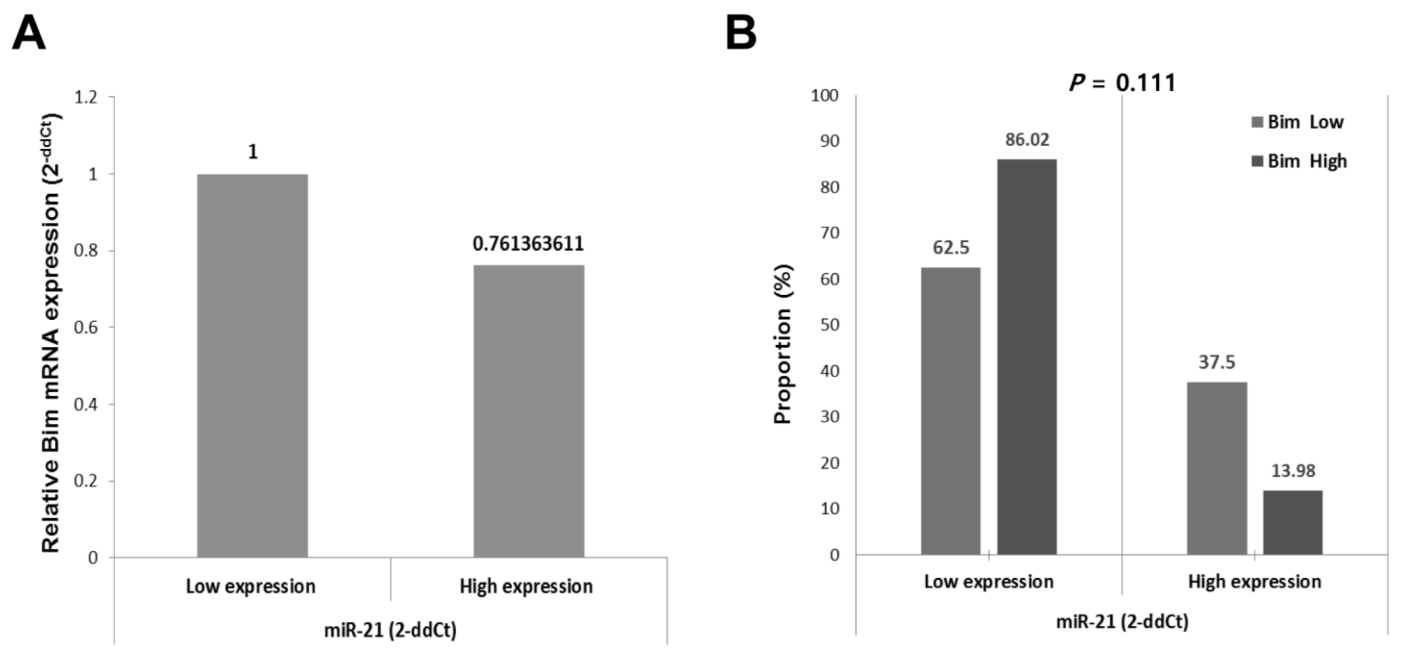

Supplementary Figure S4. Bim expression between miR-21 low and high groups of patients with DLBCL. A, Mean value of Bim mRNA expression was measured by qRT-PCR in 32 patients. B, protein expression was evaluated by IHC in 101 patients. The relationship between the Bim-IHC expression and the levels of miR-21 expression was analyzed using the Fisher's exact test.

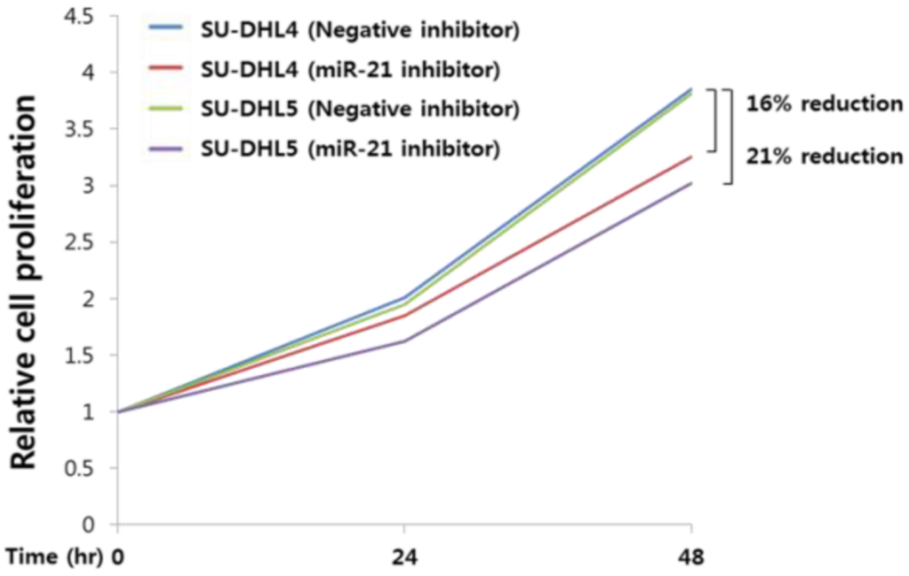

Supplementary Figure S5. After SU-DHL4 and SU-DHL5 were transfected with a miR-21 inhibitor or negative inhibitor, their cell viability and proliferation rates were determined using the CCK8 assay at 24 and 48 hours.

A

miR-21 binding motifs (5'-AGCUUAU-3')

B

FOXO1 binding motifs (5'-GTAAACAA-3')

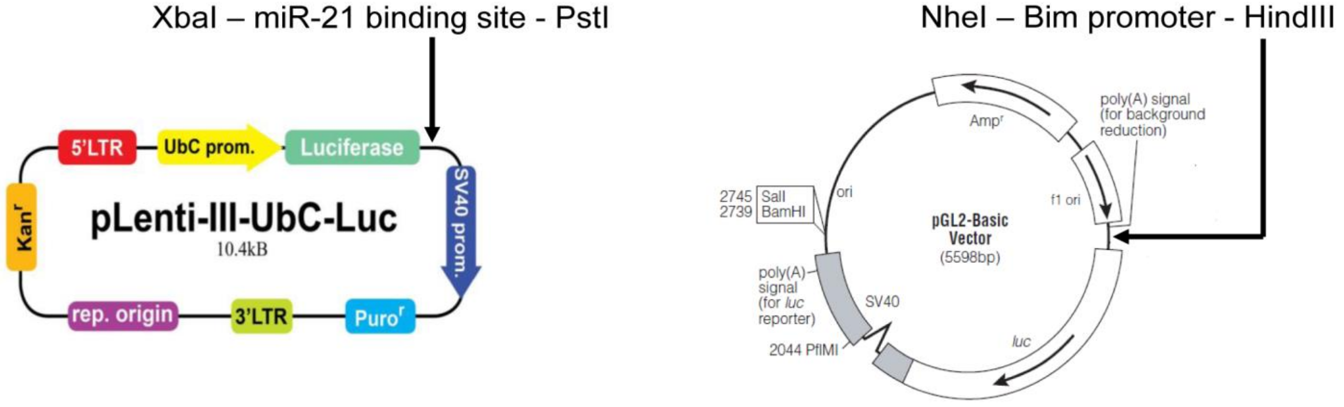

Supplementary Figure S6. A, FOXO1 3' UTR-reporter gene assay construct. B, Bim promoter-reporter gene assay construct.
